# Research on drinking water purification technologies for household use by reducing total dissolved solids (TDS)

**DOI:** 10.1371/journal.pone.0257865

**Published:** 2021-09-28

**Authors:** Bill B. Wang

**Affiliations:** Redlands East Valley High School, Redlands, California, United States of America; Mohanlal Sukhadia University, INDIA

## Abstract

This study, based in San Bernardino County, Southern California, collected and examined tap water samples within the area to explore the feasibility of adopting non-industrial equipment and methods to reduce water hardness and total dissolved solids(TDS). We investigated how water quality could be improved by utilizing water boiling, activated carbon and sodium bicarbonate additives, as well as electrolysis methods. The results show that heating is effective at lower temperatures rather than long boils, as none of the boiling tests were lower than the original value. Activated carbon is unable to lower TDS, because it is unable to bind to any impurities present in the water. This resulted in an overall TDS increase of 3.5%. However, adding small amounts of sodium bicarbonate(NaHCO_3_) will further eliminate water hardness by reacting with magnesium ions and improve taste, while increasing the pH. When added to room temperature tap water, there is a continuous increase in TDS of 24.8% at the 30 mg/L mark. The new findings presented in this study showed that electrolysis was the most successful method in eliminating TDS, showing an inverse proportion where an increasing electrical current and duration of electrical lowers more amounts of solids. This method created a maximum decrease in TDS by a maximum of 22.7%, with 3 tests resulting in 15.3–16.6% decreases. Furthermore, when water is heated to a temperature around 50°C (122°F), a decrease in TDS of around 16% was also shown. The reduction of these solids will help lower water hardness and improve the taste of tap water. These results will help direct residents to drink more tap water rather than bottled water with similar taste and health benefits for a cheaper price as well as a reduction on plastic usage.

## Introduction

The concentration of total dissolved solids(TDS) present in water is one of the most significant factors in giving water taste and also provides important ions such as calcium, magnesium, potassium, and sodium [1–3]. However, water with high TDS measurements usually indicates contamination by human activities, such as soil and agricultural runoff caused by irrigation, unregulated animal grazing and wildlife impacts, environmentally damaging farming methods such as slash and burn agriculture, and the overuse of nitrate-based fertilizer [4,5], etc. Around tourist areas as well as state parks, these factors will slowly add up over time and influence the water sources nearby [5]. Water that flows through natural springs and waterways with high concentrations of organic salts within minerals and rocks, or groundwater that originates from wells with high salt concentration will also result in higher particle measurements [6].

Water sources can be contaminated by substances and ions such as nitrate, lead, arsenic, and copper [7,8] and may cause many health problems related to heavy metal consumption and poisoning. Water reservoirs and treatments plants that do not consider water contamination by motor vehicles, as well as locations that struggle to provide the necessary components required for water treatment will be more prone to indirect contamination [9–11]. Many plants are effective in ensuring the quality and reduction of these contaminants, but often leave out the secondary considerations, The United States Environmental Protection Agency(US EPA)’s secondary regulations recommend that TDS should be below 500 mg/L [2], which is also supported by the World Health Organization(WHO) recommendation of below 600 mg/L and an absolute maximum of less than 1,000 mg/L [3]. These substances also form calcium or magnesium scales within water boilers, heaters, and pipes, causing excess buildup and drain problems, and nitrate ions may pose a risk to human health by risking the formation of *N*-nitroso compounds(NOC) and less public knowledge about such substances [12–15]. Nitrates can pose a non-carcinogenic threat to different communities, but continue to slip past water treatment standards [15]. Furthermore, most people do not tolerate or prefer water with high hardness or chlorine additives [16], as the taste changes tremendously and becomes unpreferable. Even so, TDS levels are not accounted for in mandatory water regulations, because the essential removal of harmful toxins and heavy metals is what matters the most in water safety. Some companies indicate risks in certain ions and alkali metals, showing how water hardness is mostly disregarded and is not as well treated as commercial water bottling companies [17,18].

In Southern California, water quality is not as well maintained than the northern counties as most treatment plants in violation of a regulation or standard are located in Central-Southern California [19], with southern counties having the largest number of people affected [20]. This study is focused on the Redlands area, which has had no state code violations within the last decade [21]. A previous study has analyzed TDS concentrations throughout the Santa Ana Basin, and found concentrations ranging from 190–600 ppm as treated wastewater and samples obtained from mountain sites, taking into account the urban runoff and untreated groundwater as reasons for elevated levels of TDS but providing no solution in helping reduce TDS [22]. Also, samples have not been taken directly through home water supplies, where the consumer is most affected. Other water quality studies in this region have been focused on the elimination of perchlorates in soil and groundwater and distribution of nitrates, but such research on chemicals have ceased for the last decade, demonstrated by safe levels of perchlorates and nitrates in water reports [23,24]. In addition to these studies, despite the improving quality of the local water treatment process, people prefer bottled water instead of tap water because of the taste and hardness of tap water [25]. Although water quality tests are taken and documented regularly, the taste of the water is not a factor to be accounted for in city water supplies, and neither is the residue left behind after boiling water. The residue can build up over time and cause appliance damage or clogs in drainage pipes.

This study will build upon previous analyses of TDS studies and attempt to raise new solutions to help develop a more efficient method in reducing local TDS levels, as well as compare current measurements to previous analyses to determine the magnitude to which local treatment plants have improved and regulated its treatment processes.

Several methods that lower TDS are reviewed: boiling and heating tap water with and without NaHCO₃, absorption by food-grade activated carbon [26,27], and battery-powered electrolysis [28–30]. By obtaining water samples and determining the difference in TDS before and after the listed experiments, we can determine the effectiveness of lowering TDS. The results of this study will provide options for residents and water treatment plants to find ways to maintain the general taste of the tap water, but also preserve the lifespan of accessories and pipelines. By determining a better way to lower TDS and treat water hardness, water standards can be updated to include TDS levels as a mandatory measurement.

## Materials and methods

All experiments utilized tap water sourced from Redlands homes. This water is partially supplied from the Mill Creek (Henry Tate) and Santa Ana (Hinckley) Water Sheds/Treatment Plants, as well as local groundwater pumps. Water sampling and sourcing were done at relatively stable temperatures of 26.9°C (80.42°F) through tap water supplies. The average TDS was measured at 159 ppm, which is slightly lower than the reported 175 ppm by the City of Redlands. Permission is obtained by the author from the San Bernardino Municipal Water Department website to permit the testing procedures and the usage of private water treatment devices for the purpose of lowering water hardness and improving taste and odor. The turbidity was reported as 0.03 Nephelometric Turbidity Units (NTU) post-treatment. Residual nitrate measured at 2.3mg/L in groundwater before treatment and 0.2 mg/L after treatment and perchlorate measured at 0.9 μg/L before treatment, barely staying below the standard of 1 μg/L; it was not detected within post-treatment water. Lead content was not detected at all, while copper was detected at 0.15 mg/L.

For each test, all procedures were done indoors under controlled temperatures, and 20 L of fresh water was retrieved before each test. Water samples were taken before each experimental set and measured for TDS and temperature, and all equipment were cleaned thoroughly with purified water before and after each measurement. TDS consists of inorganic salts and organic material present in solution, and consists mostly of calcium, magnesium, sodium, potassium, carbonate, chloride, nitrate, and sulfate ions. These ions can be drawn out by leaving the water to settle, or binding to added ions and purified by directly separating the water and ions. Equipment include a 50 L container, 1 L beakers for water, a graduated cylinder, a stir rod, a measuring spoon, tweezers, a scale, purified water, and a TDS meter. A standard TDS meter is used, operated by measuring the conductivity of the total amount of ionized solids in the water, and is also cleaned in the same manner as aforementioned equipment. The instrument is also calibrated by 3 pH solutions prior to testing.All results were recorded for and then compiled for graphing and analysis.

### Heating/Boiling water for various lengths of time

The heating method was selected because heat is able to break down calcium bicarbonate into calcium carbonate ions that are able to settle to the bottom of the sample. Four flasks of 1 L of tap water were each heated to 40°C, 50°C, 60°C, and 80°C (104–176°F) and observed using a laser thermometer. The heated water was then left to cool and measurements were made using a TDS meter at the 5, 10, 20, 30, and 60-minute marks.

For the boiling experiments, five flasks of 1 L of tap water were heated to boil at 100°C (212°F). Each flask, which was labeled corresponding to its boiling duration, was marked with 2, 4, 6, 10, and 20 minutes. Each flask was boiled for its designated time, left to cool under open air, and measurements were made using a TDS meter at the 5, 10, 20, 30, 60, and 120-minute marks. The reason that the boiling experiment was extended to 120 minutes was to allow the water to cool down to room temperature.

### Activated carbon as a water purification additive

This test was performed to see if food-grade, powdered activated carbon had any possibility of binding with and settling out residual particles. Activated carbon was measured using a milligram scale and separated into batches of 1, 2, 4, 5, 10, 30, and 50 mg. Each batch of the activated carbon were added to a separate flask of water and stirred for five minutes, and finally left to settle for another five minutes. TDS measurements were recorded after the water settled.

### Baking soda as a water purification additive

To lower scale error and increase experimental accuracy, a concentration of 200 mg/L NaHCO₃ solution was made with purified water and pure NaHCO₃. For each part, an initial TDS measurement was taken before each experiment.

In separate flasks of 1 L tap water, each labeled 1, 2, 4, 5, 10, and 30 mg of NaHCO_3_, a batch was added to each flask appropriately and stirred for 5 minutes to ensure that everything dissolved. Measurements were taken after the water was left to settle for another 5 minutes for any TDS to settle.

Next, 6 flasks of 1 L tap water were labeled, with 5 mg (25 mL solution) of NaHCO₃ added to three flasks and 10 mg (50 mL solution) of NaHCO₃ added to the remaining three. One flask from each concentration of NaHCO₃ was boiled for 2 mins., 4 mins., or 6 mins., and then left to cool. A TDS measurement was taken at the 5, 10, 20, 30, 60, and 120-minute marks after removal from heat.

### Electrolysis under low voltages

This test was performed because the ionization of the TDS could be manipulated with electricity to isolate an area of water with lower TDS. For this test, two 10cm long graphite pieces were connected via copper wiring to a group of batteries, with each end of the graphite pieces submerged in a beaker of tap water, ~3 cm apart.

Using groups of 1.5 V double-A batteries, 4 beakers with 40mL of tap water were each treated with either 7.5, 9.0, 10.5, and 12.0 V of current. Electrolysis was observed to be present by the bubbling of the water each test, and measurements were taken at the 3, 5, 7, and 10 minute marks.

## Results/Discussion

### Heating water to various temperatures until the boiling point

The goal for this test was to use heat to reduce the amount of dissolved oxygen and carbon dioxide within the water, as shown by this chemical equation: Heat: Ca(HCO_3_)_2_ → CaCO_3_↓ + H_2_O + CO_2_↑.

This would decompose ions of calcium bicarbonate down into calcium carbonate and water and carbon dioxide byproducts.

#### Patterns and trends in decreasing temperatures

**Fig 1:**
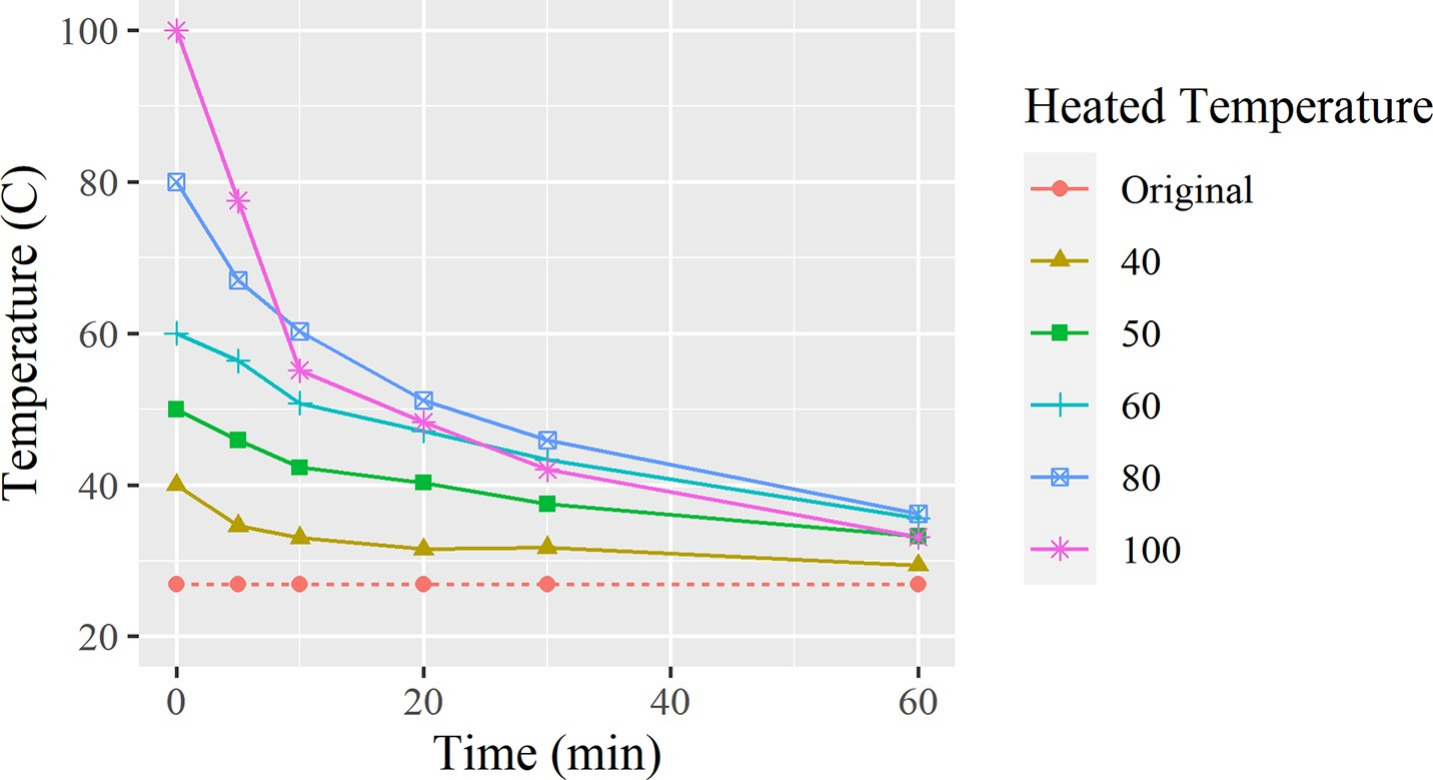
Temperature change overtime for each starting temperature.

The following trend lines are based on a dataset of changes in temperature obtained from the test results and graphed as Fig 1.

To predict the precise temperature measurements of the tap water at 26.9°C, calculations were made based on Fig 1. The fitting equations are in the format, y = a.e^bx^. The values for the fitting coefficients a and b, and correlation coefficient R^2^ are listed in Table 1 as column a, b and R^2^. The calculated values and the target temperature are listed in Table 1.

**Table 1:** Calculated durations to reach the target temperature of 26.9°C.

Heating Temperature (°C)	a	b	R^2^	Duration to Target Temperature (min)	Target Temperature (°C)
40	36.07	-0.004	0.6798	73.3	26.90
50	46.978	-0.006	0.9175	92.9	26.90
60	57.43	-0.008	0.9639	94.8	26.90
80	71.066	-0.012	0.9175	80.95	26.90
100	78.163	-0.017	0.8101	62.75	26.90

**Fig 2:**
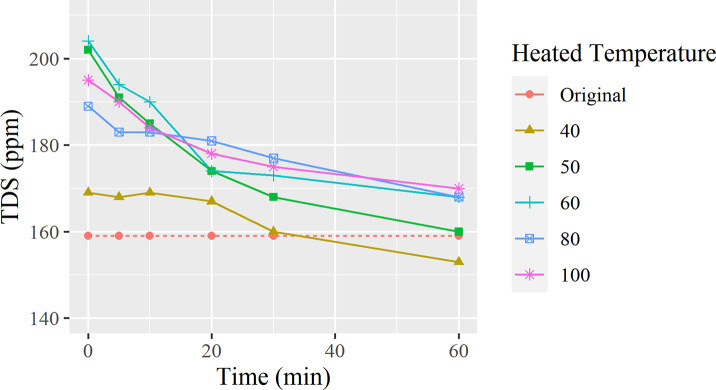
TDS change overtime for each starting temperature.

Fig 2 was obtained by compiling TDS results with different temperatures and times.

The fitting equations for Fig 2 are also in the format, y = a.e^bx^. The fitting coefficients a and b, and correlation coefficient R^2^ values are listed in Table 2. Based on the fitting curves in Fig 2 and the duration to the target temperature in Table 1, We calculated the TDS at 26.9°C as listed in column calculated TDS in Table 2 based on the values we reported on Fig 2.

**Table 2:** Calculated TDS measurements at 26.9°C.

Heating Temperature (°C)	a	b	R^2^	Duration to Target Temperature (min)	Calculated TDS (PPM)
40	170.4	-0.002	0.9363	73.3	147.17
50	193.65	-0.004	0.8687	92.9	133.55
60	193.46	-0.003	0.7675	94.8	145.58
80	186.86	-0.002	0.9628	80.95	158.93
100	190.2	-0.002	0.849	62.75	167.77

Based on the heating temperature and the calculated TDS with the same target water temperature, we obtained the following heating temperature vs TDS removal trend line and its corresponding fitting curve in Table 2.

In Fig 1, a trend in the rate of cooling is seen, where a higher heating temperature creates a steeper curve. During the first five minutes of cooling, the water cools quicker as the absorbed heat is quickly released into the surrounding environment. By the 10-minute mark, the water begins to cool in a linear rate of change. One detail to note is that the 100°C water cools quicker than the 80°C and eventually cools even faster than the 60°C graph. Table 1 supports this observation as the duration to target temperature begins to decrease from a maximum point of 94.8 mins to 80.95 mins after the 80°C mark.

As shown in Fig 2, all TDS values decrease as the temperature starts to cool to room temperature, demonstrating a proportional relationship where a lower temperature shows lower TDS. This can partially be explained by the ions settling in the flasks. Visible particles can also be observed during experimentation as small white masses on the bottom, as well as a thin ring that forms where the edge of the water contacts the flask. When the water is heated to 40°C and cooled, a 3.8% decrease in TDS is observed. When 50°C is reached, the TDS drops at its fastest rate from an initial value of 202 ppm to 160 ppm after 60 minutes of settling and cooling. The TDS measurements in these experiements reach a maximum of 204 ppm at the 60°C mark. However, an interesting phenomenon to point out is that the water does not hit a new maximum at 100°C. meaning that TDS reaches a plateau at 60°C. Also, the rate of decrease begins to slow down after 20 minutes, showing that an unknown factor is affecting the rate of decrease. It is also hypothesized that the slight increase in TDS between the 5–20 minute range is caused by a disturbance in the settling of the water, where the temperature starts to decrease at a more gradual and constant rate. The unstable and easy formation of CaCO_3_ scaling has also been the subject of a study of antiscaling methods, which also supports the result that temperature is a significant influence for scale formation [12].

In Table 2, calculations for TDS and the time it takes for each test to cool were made. Using the data, it is determined that the test with 50°C water decreased the most by 16% from the initial measurement of 159ppm. This means that it is most effective when water is heated between temperatures of 40–60°C when it comes to lowering TDS, with a difference of ~7–16%. When water is heated to temperatures greater than 80°C, the water begins to evaporate, increasing the concentration of the ions, causing the TDS to increase substantially when cooled to room temperature.

**Fig 3:**
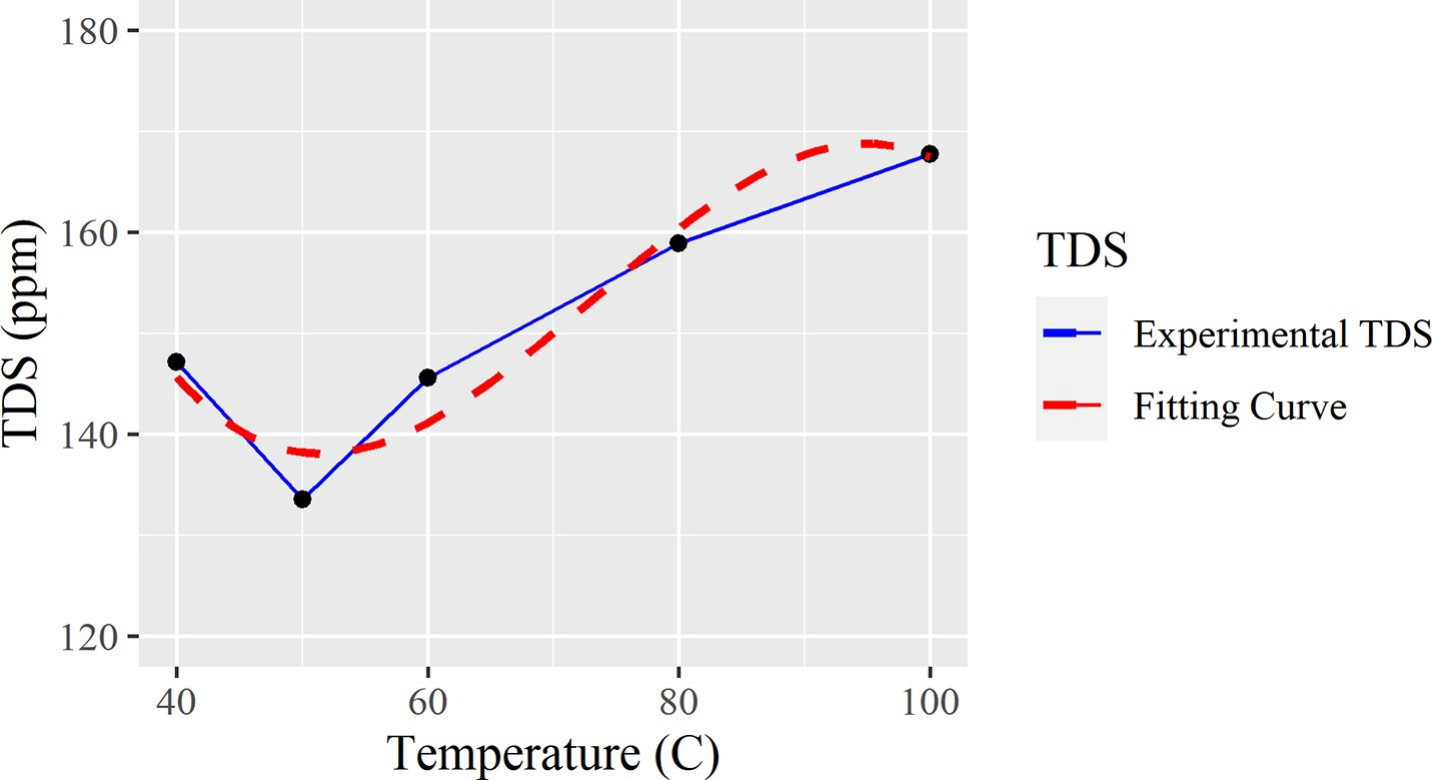
Fitting curve of TDS changes over all temperatures between 40–100°C.

Finally, in Fig 3, a line of best fit of function f(x) = -0.0007x^3^ + 0.1641x^2^–10.962x + 369.36 is used with R^2^ = 0.9341. Using this function, the local minimum of the graph would be reached at 48.4°C.

This data shows that heating water at low temperatures (i.e. 40–50°C) may be more beneficial than heating water to higher temperatures. This study segment has not been presented in any section within the United States EPA Report on water management for different residual particles/substances. However, warmer water temperatures are more prone to microorganism growth and algal blooms, requiring more intensive treatment in other areas such as chlorine, ozone, and ultraviolet disinfection.

Using the specific heat capacity equation, we can also determine the amount of energy and voltage needed to heat 1 L of water up to 50°C: Q = mcΔT, where c, the specific heat capacity of water, is 4.186 J/g°C, ΔT, the change in temperature from the experimental maximum to room temperature, is 30°C, and m, the mass of the water, is 1000 g. This means that the amount of energy required will be 125580 J, which is 0.035 kWh or 2.1 kW.

**Fig 4:**
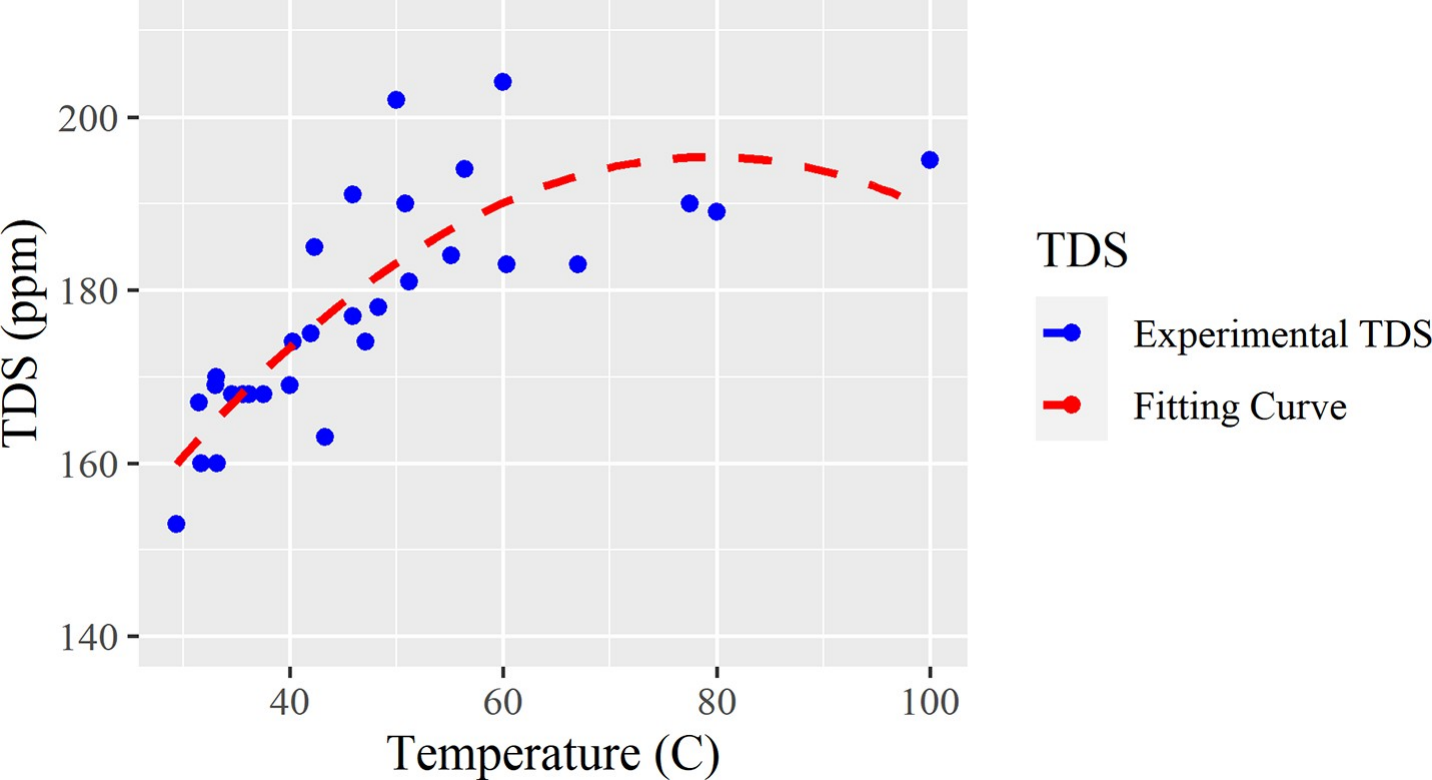
Scatterplot and a fitting curve of TDS under different temperatures.

After taking all of the different measurements obtained during TDS testing, and compiling the data onto this plot, Fig 4 is created with a corresponding line of best fit:

In Fig 4, it can be observed that the relationship between the temperature of the water and its relative TDS value is a downwards facing parabolic graph. As the temperature increases, the TDS begins to decrease after the steep incline at 50–60°C. The line of best fit is represented by the function f(x) = -0.0142x^2^ + 2.258x + 105.84. R^2^ = 0.6781. Because the R^2^ value is less than expected, factors such as the time spent settling and the reaction rate of the ions should be considered. To determine the specifics within this experiment, deeper research and prolonged studies with more highly accurate analyses must be utilized to solve this problem.

### Boiling water for various amounts of time

#### Trend of boiling duration and rate of cooling

**Fig 5:**
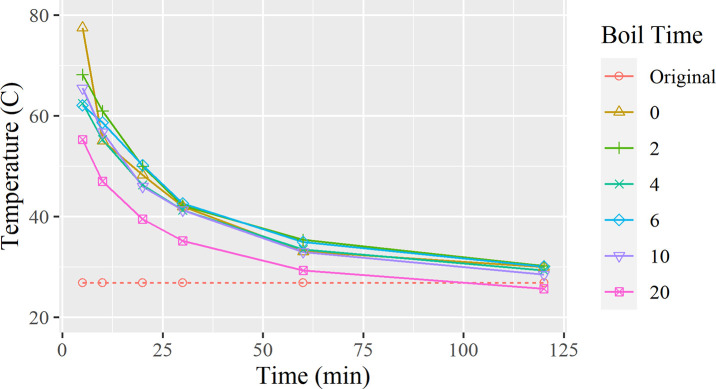
Water temperature change overtime organized by corresponding boiling durations.

Using the same methods to create the figures and tables for the previous section, Fig 5 depicts how the duration of time spent boiling water affects how fast the water cools.

As seen in Fig 5, within the first 10 minutes of the cooling time, the five different graphs are entwined with each other, with all lines following a similar pattern. However, the graph showing 20 minutes of boiling is much steeper than the other graphs, showing a faster rate of cooling. This data continues to support a previous claim in Fig 2, as this is most likely represented by a relationship a longer the boil creates a faster cooling curve. This also shows that the first 5 minutes of cooling have the largest deviance compared to any other time frame.

The cooling pattern is hypothesized by possible changes in the orderly structure of the hydrogen bonds in the water molecules, or the decreased heat capacity of water due to the increasing concentration of TDS.

#### Effect on TDS as boiling duration increases

**Fig 6:**
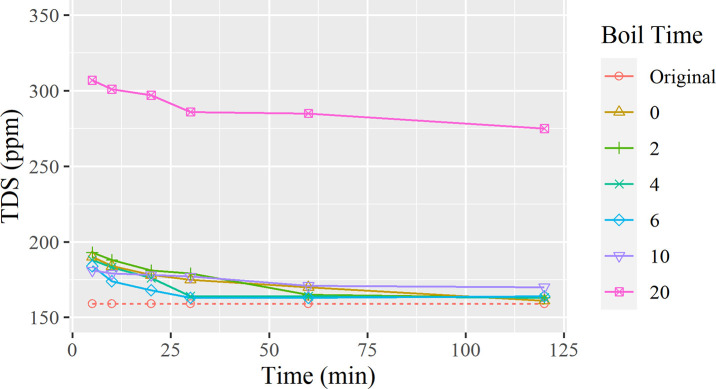
TDS change overtime organized by corresponding boiling durations.

In Fig 6, all lines except for the 20-minute line are clustered in the bottom area of the graph. By excluding the last measurement temporarily due to it being an outlier, we have observed that the difference between the initial and final TDS value of each test decreases.

Despite following a similar trend of an increase in TDS at the start of the tests and a slow decrease overtime, this experiment had an interesting result, with the final test measuring nearly twice the amount of particles compared to any previous tests at 310 ppm, as shown in Fig 6. It is confirmed that the long boiling time caused a significant amount of water to evaporate, causing the minerals to be more concentrated, thus resulting in a 300 ppm reading. Fig 6 follows the same trend as Fig 2, except the TDS reading veers away when the boiling duration reaches 20 minutes. Also, with the long duration of heating, the water has developed an unfavorable taste from intense concentrations of CaCO₃. This also causes a buildup of a thin crust of CaCO₃ and other impurities around the container that is difficult to remove entirely. This finding is in accordance with the introductory statement of hot boiling water causing mineral buildups within pipes and appliances [9]. A TDS reading of 300ppm is still well below federal secondary standards of TDS, and can still even be compared to bottled water, in which companies may fluctuate and contain 335ppm within their water [1,2].

This experiment continues to stupport that the cooling rate of the water increases as the time spent boiling increases. Based on this test, a prediction can be made in which an increased concentration of dissolved solids lowers the total specific heat capacity of the sample, as the total volume of water decreases. This means that a method can be derived to measure TDS using the heat capacity of a tap water mixture and volume, in addition to current methods of using the electrical conductivity of aqueous ions.

### Adding food-grade activated carbon to untreated tap water

**Fig 7:**
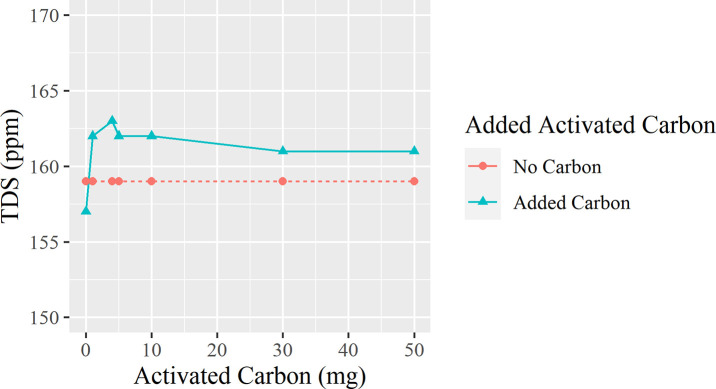
Trend for the effect of activated carbon on TDS.

Fig 7 presents a line graph with little to no change in TDS, with an initial spike from 157 to 163 ppm. The insoluble carbon remains in the water and shows no benefit.

The food-grade activated carbon proved no benefit to removing TDS from tap water, and instead added around 5–7 ppm extra, which settled down to around +4 ppm at 120 minutes. The carbon, which is not 100% pure from inorganic compounds and materials present in the carbon, can dissolve into the water, adding to the existing concentration of TDS. Furthermore, household tap water has already been treated in processing facilities using a variety of filters, including carbon, so household charcoal filters are not effective in further reducing dissolved solids [18].

### Adding sodium bicarbonate solution to boiled tap water

**Fig 8:**
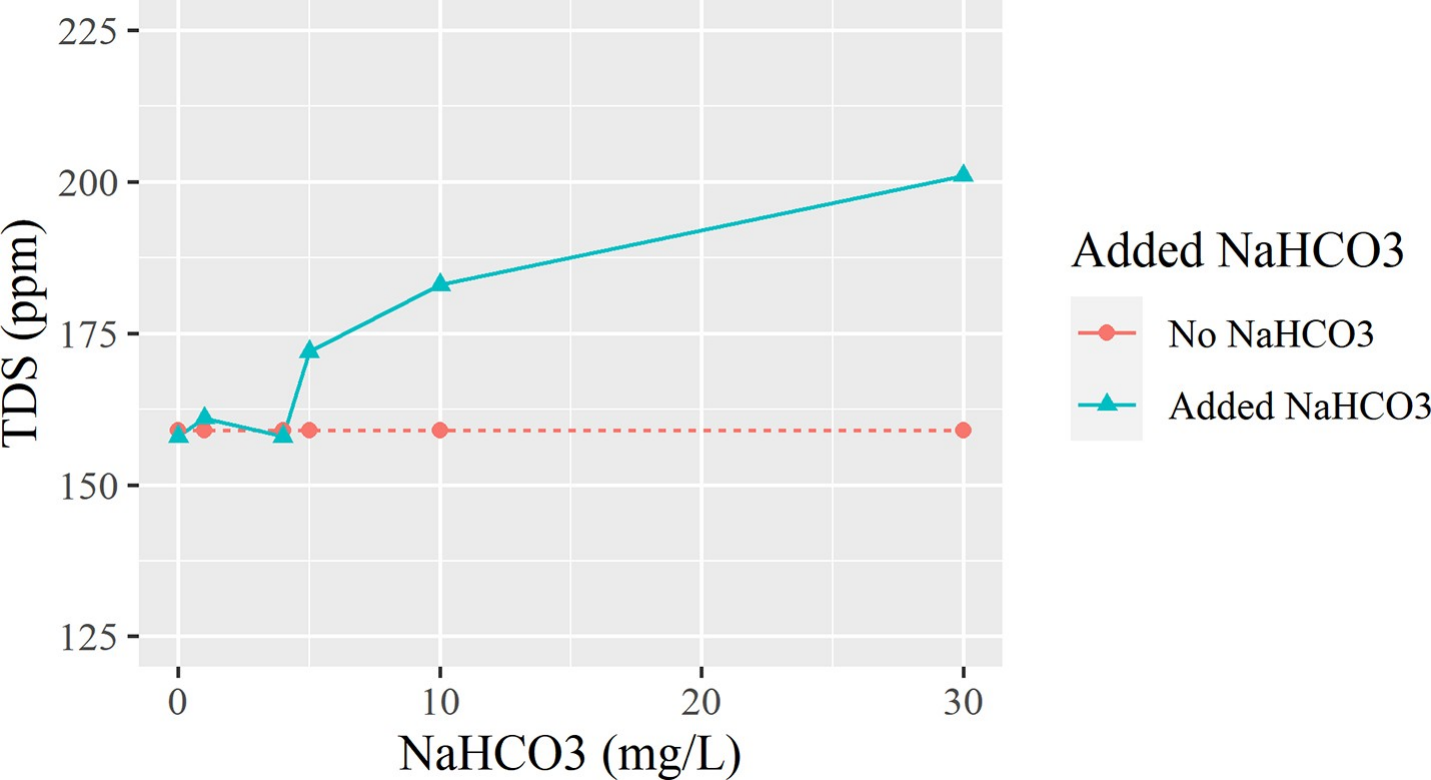
Trend for the effect of NaHCO3 on TDS.

As seen in Fig 8, after adding 1 mg of NaHCO_3_ in, the TDS rises to 161 ppm, showing a minuscule increase. When 4 mg was added, the TDS drops down to 158 ppm. Then, when 5 mg was added, a sudden spike to 172 ppm was observed. This means that NaHCO_3_ is able to ionize some Ca^2+^ and Mg^2+^ ions, but also adds Na^+^ back into the water. This also means that adding NaHCO_3_ has little to no effect on TDS, with 4mg being the upper limit of effectiveness.

**Fig 9:**
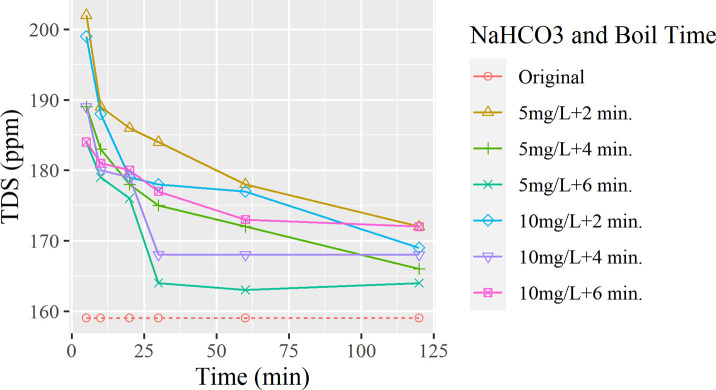
Trend for the effect of NaHCO3 on boiling water TDS.

To examine whether or not the temperature plays a role in the effectiveness in adding NaHCO_3_, a boiling experiment was performed, and the data is graphed in Fig 9.

Fig 9 presents the relationship between the amount of common baking soda(NaHCO₃) added, the boiling time involved, and the resulting TDS measurements. After boiling each flask for designated amounts of time, the results showed a downward trend line from a spike but does not reach a TDS value significantly lower than the initial sample. It is apparent that the NaHCO₃ has not lowered the TDS of the boiling water, but instead adds smaller quantities of ions, raising the final value. This additive does not contribute to the lowering of the hardness of the tap water. However, tests boiled with 5 mg/L of baking soda maintained a downward pattern as the water was boiled for an increasing amount of time, compared to the seemingly random graphs of boiling with 10 mg/L.

In some households, however, people often add NaHCO₃ to increase the pH for taste and health benefits. However, as shown in the test results, it is not an effective way of reducing TDS levels in the water [10,16], but instead raises the pH, determined by the concentration added. Even under boiling conditions, the water continues to follow the trend of high growth in TDS, of +25–43 ppm right after boiling and the slow drop in TDS (but maintaining a high concentration) as the particles settle to the bottom.

Utilizing the experimental results, we can summarize that after adding small batches of NaHCO3 and waiting up to 5 minutes will reduce water hardness making it less prone to crystallizing within household appliances such as water brewers. Also, this process raises the pH, which is used more within commercial water companies. However, the cost comes at increasing TDS.

### Using electrolysis to treat TDS in tap water

**Fig 10:**
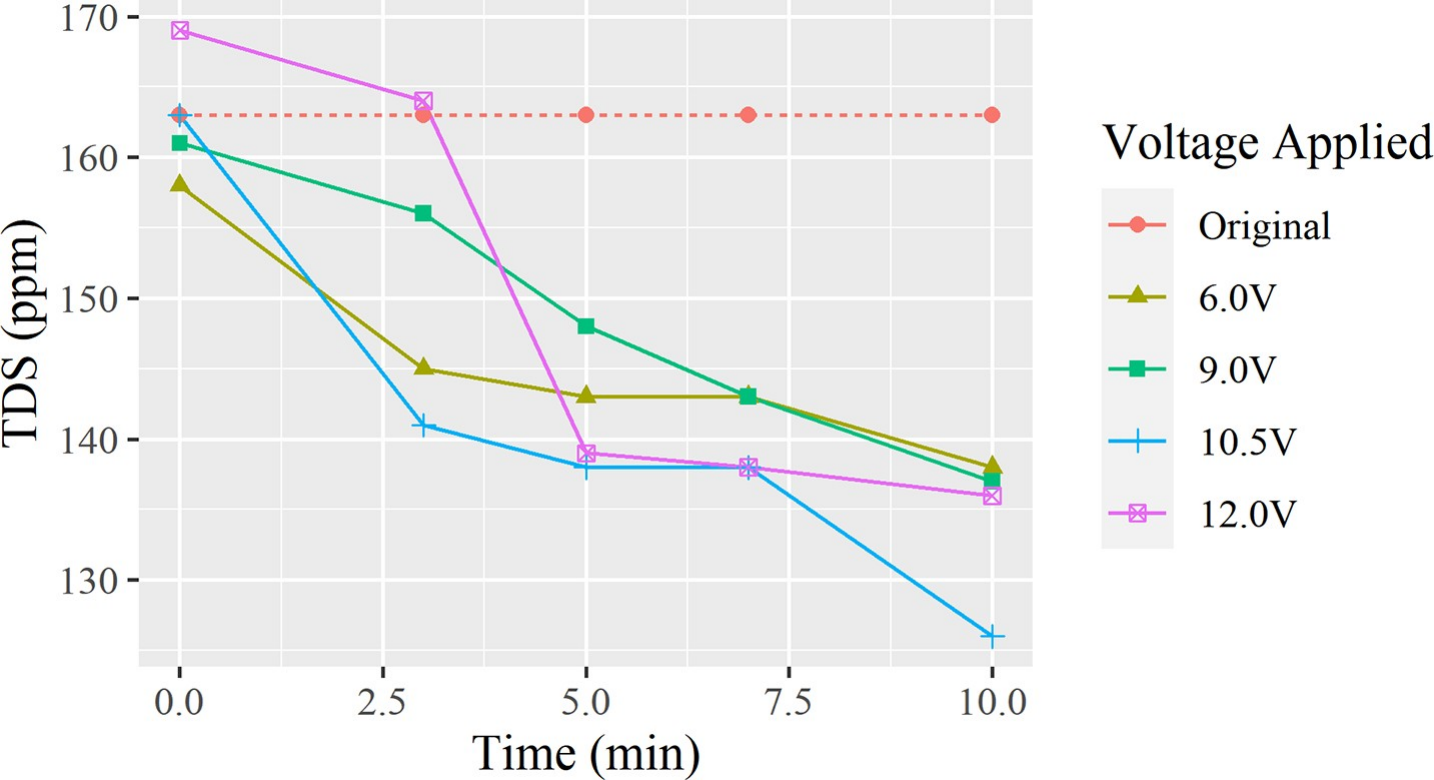
TDS changes with different electrical voltages.

Different voltages were passed through the water to observe the change in TDS overtime, with the data being compiled as Fig 10.

The process of electrolysis in this experiment was not to and directly remove the existing TDS, but to separate the water sample into three different areas: the anode, cathode, and an area of clean water between the two nodes [19]. The anions in the water such as OH^-^, SO_4_^2-^, HCO_3_^-^ move to the anode, while the cations such as H^+^, Ca^2+^、Mg^2+^、Na^+^ move to the cathode. The middle area would then be left as an area that is more deprived of such ions, with Fig 10 proving this.

As shown in Fig 10, electrolysis is effective in lower the TDS within tap water. Despite the lines being extremely tangled and unpredictable, the general trend was a larger decrease with a longer duration of time. At 10 minutes, all lines except 10.5 V are approaching the same value, meaning that the deviation was most likely caused by disturbances to the water during measurement from the low volume of water. With each different voltage test, a decrease of 12.7% for 6.0 V, 14.9% for 9.0 V, 22.7% for 10.5 V. and 19.5% for 12.0 V respectfully were observed. In the treatment of wastewate leachate, a study has shown that with 90 minutes of electrical treatment, 34.58% of TDS content were removed, supporting the effectiveness of electricity and its usage in wastewater treatment [29].

This experiment concludes that electrolysis is effective in lowering TDS, with the possibility to improve this process by further experimentation, development of a water cleaning system utilizing this cathode-anode setup to process water. This system would be a more specific and limited version of a reverse osmosis system by taking away ions through attraction, rather than a filter.

## Conclusion

The Southern Californian tap water supply maintains TDS values below the federal regulations. However, crystalline scale buildup in household appliances is a major issue as it is hard to clean and eliminate. To easily improve the taste and quality of tap water at home as well as eliminating the formation of scales, the following methods were demonstrated as viable:

1. By heating water to around 50°C (122°F), TDS and water hardness will decrease the most. Also, the boiling process is effective in killing microorganisms and removing contaminants. This process cannot surpass 10 minutes, as the concentration of the ions in the water is too high, which poses human health risks if consumed. These, along with activated carbon and NaHCO₃ additives, are inefficient methods that have minimal effects for lowering TDS.
2. Electrolysis is one of the most effective methods of eliminating TDS. Experiments have proven that increased current and duration of time helps lower TDS. However, this method has yet to be implemented into conventional commercial water filtration systems.

Also, some observations made in these experiments could not be explained, and require further research and experimentation to resolve these problems. The first observation is that TDS and increasing water temperature maintain a parabolic relationship, with a maximum being reached at 80°C, followed by a gradual decrease. The second observation is that when water is boiled for an increased duration of time, the rate of cooling also increases.

This experiment utilized non-professional scientific equipment which are prone to mistakes and less precise. These results may deviate from professionally derived data, and will require further study using more advanced equipment to support these findings.
